# Progress in Disease of Digestive Organs

**Published:** 1900-12-15

**Authors:** 


### Progress in Disease of Digestive Organs.

**Colitis.**—Mathieu [25] in speaking of membranous colitis lays stress on the need of removing the constipation which always accompanies it. He gives small doses of castor oil, belladonna, and large enemata containing mild antiseptics. Boas, too, remarks on its connection with constipation, and notes that one form may be produced by astringent injections in weakly persons. Mannaberg distinguishes paroxysmal mucous colic from membranous colitis. The latter is a mere catarrh, the former follows some neurosis of which it is probably only a local symptom. W. H. Thompson,[26] however, holds that it is no more a neurosis than a gleet is, since nervous depression frequently accompanies a gonorrhœa. He gives copious hot saline injections with five drops of oil of peppermint to the pint once or twice a day, as well as small repeated doses of castor-oil for months. Nitrate of silver and turpentine may be taken in capsules. Crowder [27] discusses the rare condition, chronic hyperplastic tuberculosis of the cæcum. There is great thickening and contraction of the bowel which may be mistaken for carcinoma. The mucous membrane is beset with little papillomatous masses around the ulcers, sometimes pedunculated and polypoid. These chronic affections of the cæcum seem to be secondary to slight or healed pulmonary troubles, usually lead to obstruction of the bowel, and may become the seat of cancerous growth.

**Appendicitis.**—The editor of the *Medical Record* [28] remarks that there are three views as to operation. Some insist it should take place as soon as the diagnosis is made, others wait till there is a special reason at a favourable season, and some wait as long as possible, believing that many cases do not recur. Speaking according to the received view in America he holds that the last course is untenable. E. G. Janeway [29] enumerates among conditions liable to be mistaken for appendicitis—neuralgias, reflected pains of pulmonary disease, renal colic, hydronephrosis, floating kidney, cholecystitis, gastric and other ulcers, colic, tubercular ulcers, cancer of the cæcum, fæcal impaction with or without stricture of the colon, typhoid, abscess of the ovary, the general pains of tonsillitis, and retroperitoneal abscess. Marchant [30] adds—inflammation of glands before or behind the cæcum. Rosewater [31] in early stages of the disease would give calomel and enemata of salt water, and keep an ice-bag on the spot. If vomiting is troublesome at this stage $\frac{1}{20}$ of a grain of calomel every hour may be tried, and in convalescence iodide of ammonium to release the intestinal adhesions. He administers copious draughts of hot water throughout the entire attack. Subphrenic abscess has followed appendicitis in a few instances. T. F. Baldwin [32] reports two more cases, making 45 on record. Both instances occurred in women convalescents after the removal of an appendix. They may be either intra or extra-peritoneal. Usually there is a return of general symptoms with tenderness in the hepatic region, and perhaps pain in the right shoulder, hiccough, sometimes invasion of the pleural cavity, or there may be gas in the abscess cavity with amphoric breath sounds over it. These abscesses probably occur often though unrecognised, since B. Robinson [33] shows that solid particles in the peritoneum tend to be absorbed chiefly through the lymphatics of the diaphragm rather than in other parts of its area. In five minutes after injection of Berlin blue into the peritoneum the granules can be found in these lymphatics. If leucocytes do not suffice in destroying an infection, and exudation does not dam it off and surround it so as to exclude the rest of the peritoneum, in other words, unless a conservative peritonitis takes place, septic elements in the peritoneum are rapidly fatal.

**Liver.**—The pathology of cirrhosis was discussed at the meeting of the British Medical Association. Voelcker,[34] in speaking of the distinction between the atrophic and hypertrophic classes, laid down that there is a real distinction between the peripheral and intercellular forms. He thinks, too, that the primary change affects both the cells and the connective tissue, and that only subsequently is a variable amount of new fibrous tissue formed. Flexner showed evidence that the cells are first attacked, and proved that not only is there a new growth of fibrous tissue, but also of elastic, which had not been suspected previously. Lazarus-Barlow, too, holds that the first event is the destruction of cells, after which an overgrowth of fibrous tissue takes place to fill up the potential gap, but the latter is not an inflammatory change, at least, in the usual sense. As to the experimental production of cirrhosis by the injection of bacteria some fresh evidence was adduced. Hektoen claimed to have brought about cirrhosis in guinea-pigs in this way, but the results were not constant, and in other animals they were even less marked. Theodore Fisher discussed cases of sepsis where acute proliferation of interstitial tissue had occurred, but Voelcker replied that cirrhosis is rarely found after acute infectious diseases, or in children of certain ages who are liable to frequent attacks of septic diarrhœa, though if cirrhosis was usually due to sepsis these are just the cases where it should be commonest.

We may refer here to the Lumleian lectures, though they date some seven months back. In them Cheadle remarks that the prognosis differs much in the various forms of cirrhosis. In atrophic cases, when ascites appears, the end is usually not very distant, though much depends on the general state of nutrition. In hypertrophic forms the outlook is much brighter. As to treatment, he considers that diuretics and purgatives are quite useless for reducing the ascites, and tapping should be carried out early and often. We may add that among the patients attending a local hospital there were recently two women, one of whom had been tapped 100 times and the other 150, with good results. Cheadle thinks that a hard-and-fast line cannot be drawn between the atrophic and hypertrophic forms, but there is no reason to think that the atrophic form is preceded by a stage of enlargement except in the rarest instances. We do indeed often see an early engorgement or congestive swelling of a temporary kind in drunkards. The special symptoms noted by Hanot may all be absent in hypertrophic cirrhosis. Most of the cases are of alcoholic origin; ascites may be present and jaundice absent, and the course of the disease acute rather than chronic. In some of these hypertrophic forms it is probable that we have to do with mixed causes, and that the enlargement is partly due to alcoholic heart failure, though no murmurs may be present. Such cases respond better to treatment and a better prognosis may therefore be given. The syphilitic liver is difficult to distinguish during life from the other types, but there is in it often marked pain, tenderness, and pyrexia.

There is indeed a variety of perihepatitis not syphilitic in origin or due to cardiac changes, but difficult to diagnose during life. Smaltz and Webber[35] in describing a case suggest the cause may be some form of peritonitis which has affected the covering of the liver, such as that following an attack of appendicitis. The disease may commence suddenly, cause great pain and ascites, and last several years. The liver, though large and sensitive, shows no trace of cirrhosis or signs of marked portal obstruction, but is compressed by a thick fibrous coat. The relation of acute yellow atrophy to the acute types of cirrhosis is a difficult question. L. W. Findlay[36] is inclined to believe they are identical, save that in the former the cell destruction is extreme and the regeneration of liver tissue is considerable. Lisanti[37] notes another affection of the liver which may be confounded with cirrhosis or syphilis. This is mercurial poisoning, in which the liver is enlarged, tender, varying in size from day to day, and accompanied by slight jaundice and scanty high-coloured urine. If the patient is taking mercury medicinally, the condition ceases soon after the drug is left off and reappears when it is again employed, in which respect the disease, of course, differs from the large liver of tertiary syphilis.

**Ptosis of the Liver** forms a movable tumour as low or lower than the umbilicus, but the hepatic tissue is generally healthy. Treves[38] says it generally occurs in women with relaxed abdominal walls, but it is not caused by tight lacing, for no one makes a waist so high as the liver. It may give rise to no symptoms or to vague indefinite pains and cramps. Since the most fixed point of the liver is the origin of the vena cava, the posterior border is displaced least, while the anterior edge is prolonged downwards and forwards. At the same time the right end falls much more than the left. This movement about a double axis results in a great deformity of the organ, which also often shows a curious transverse groove.

Sometimes a *floating lobe* or tongue projects downwards and inwards. If derived from the quadrate lobe it is usually accompanied by a gall bladder containing calculi, but when it arises from the right lobe the gall bladder may be normal. Such a tumour can rarely be diagnosed correctly before laparotomy; even its connection with the liver may not be made out, and the liver itself may be normal in position and in every other respect. No treatment is desirable unless there are gall stones to remove, but a prolapsed liver can be supported by a form of belt with a metal plate, or even by suturing.

**Cholæmia.**—Merklen and Janot[39] showed a case of jaundice without bile in the urine. The jaundice in such instances develops slowly but persistently, and is accompanied by dyspepsia, weakness, and nervous pains. It is slight in degree, and shows most in the face and on the palms and soles of the feet. The fæces are of normal colour, but the bile can be easily found in the blood serum. The cause of the disease is unknown. In biliary colic J. H. Keay[40] asserts that the pain begins at the back, about the tenth or eleventh dorsal vertebra, then gradually passes round to the right, and often to the left hypochondrium and the rest of the abdomen, sometimes to the right nipple, but hardly ever to the right shoulder. It may, however, radiate into the limbs; slight attacks only may commence in the right hypochondrium. T. H. Marsh[41] describes two cases of Weil's disease or acute febrile jaundice. The patients were males, and complained of pains all over, especially in the calves of the legs; the liver was enlarged and tender, the temperature 102-103, the urine contained bile pigment and in one case blood, while the Widal test was negative.

**Pancreas.**—A case of hæmorrhagic pancreatitis is described by Maynard and Uhthoff.[42] The patient, a male, aged 77, was seized with sudden abdominal pain, vomiting of coffee-ground material, and became collapsed. The abdomen was tender but not distended. Death took place on the fourth day. The pancreas was enlarged, studded with hæmorrhages and patches of fat necrosis, which extended to the peritoneum. Mayo Robson[43] remarks that besides these acute forms we find subacute cases in which an abscess may develop; and chronic ones, which may be difficult to diagnose from cancer of the head of the pancreas, cancer of the liver and bile ducts, chronic catarrh or calculi in the common duct. The pain is most often felt in the middle line, and radiates towards the left side; there may sometimes be a tumour or a distension of the upper part of the abdomen, and the initial constipation frequently gives place to a fœtid diarrhœa, with whitish stools. An instance of acute pancreatitis complicating mumps is recorded by H. W. Jacob.[44] Here a tumour was felt in the situation of the pancreas, not moving with respiration, but extremely tender and the seat of violent pain. With the subsidence of the mumps the affection of the pancreas disappeared, and vomiting ceased. Lefas[45] states that in cirrhosis of the liver the pancreas often shows patches of interstitial fibrosis within the lobules, with some fatty degeneration. In Hanot's hypertrophic form pigmentary sclerosis takes place around the lobules, while in "cardiac sclerosis" the changes in the pancreas are less certain. The organ seems to be easily affected by alcohol.

[25] Brit. Med. Jour., Aug. 11. [26] Med. Rec., March 10. [27] Amer. Jour Med. Sci., June. [28] Med. Rec., Sept. 20. [29] Idem., May 26. [30] Quarterly Jour., August. [31] New York Med. Jour. [32] Colomb. Med. Jour., August [33] Med. Rec., July 28. [34] Lancet, Sept. 8. [35] Amer. Jour. Med. Sci., April. [36] Brit. Med. Jour., June 2. [37] New York Med. Jour., April 21. [38] Lancet, May 12. [39] Lancet, Aug. 11. [40] Brit. Med. Jour., April 14. [41] Brit. Med. Jour., July 14. [42] Brit. Med. Jour., June 23. [43] Lancet, June 28. [44] Brit. Med. Jour., June 23. [45] Idem. Ep.

